# Low incidence and morbidity of Epstein‐Barr virus reactivation following donor lymphocyte infusions

**DOI:** 10.1002/jha2.672

**Published:** 2023-03-13

**Authors:** Danilo Martinovic, Justin Hasenkamp, Wolfram Jung, Frank Tucholski, Jens‐Holger Maas, Gerald Georg Wulf

**Affiliations:** ^1^ Haematology and Medical Oncology University Medicine Göttingen Göttingen Germany; ^2^ Transfusion Medicine University Medicine Göttingen Göttingen Germany

**Keywords:** allogeneic hematopoietic stem cell transplantation (alloHSCT), donor lymphocyte infusion (DLI), Epstein‐Barr virus (EBV)

Dear Editor,

Epstein‐Barr virus (EBV) reactivation occurs under immunosuppression following solid organ or stem cell transplantation, leading to post‐transplantation lymphoproliferative disease (PTLD) in some patients. PTLD after allogeneic hematopoietic stem cell transplantation (alloHSCT) is donor‐derived, originating from B cells transmitted with the transplant. Here, we report EBV reactivations in eight of 151 consecutive patients receiving donor lymphocyte infusions (DLIs). The EBV reactivations occurred at a median interval of 52 days after initiation of DLI treatment, mostly following the second DLI graft. The mean level of EBV reactivation amounted to 6.3 × 10^3^ copies/ml, and this triggered preemptive Rituximab treatment in four patients. EBV reactivation regressed in all patients, with no PTLD cases observed.

DLIs are an effective form of adoptive immunotherapy for the treatment of disease recurrence after alloHSCT [1]. While the donor T cells exert the desired therapeutic effects, unmanipulated DLI grafts also contain donor B cells that may harbor EBV, which prompted this retrospective analysis approved by the University Medicine Goettingen (UMG) ethics committee. Out of 151 consecutive patients who had received a DLI from 2005 through 2020 at the UMG after informed consent, EBV reactivation, defined as measurable EBV load in peripheral blood or tissue biopsy was detected by quantitative polymerase chain reaction (PCR) testing in eight patients. Importantly, all of the patients with EBV reactivation had negative PCR findings prior to the initiation of the treatment with DLI, measured at the last follow‐up visit. Four of these patients had a history of low‐titer EBV reactivation prior to DLI treatment. In all eight cases, patients and their respective donors were EBV seropositive prior to transplantation. Peripheral blood stem cells have been used as graft‐source in all eight cases. For GVHD prophylaxis, calcineurin inhibitors in combination with mycophenolate and anti‐thymocyte globulin (ATG) were employed. At the time of the DLI, all patients had been free of immunosuppressive drugs for at least 6 weeks. One to a maximum of five DLIs were applied to treat disease recurrence in seven patients and failing chimerism in one patient respectively, at a median interval after alloHSCT of 518 days (range: 105–2350 days). The dose of DLI was determined according to the institutional standard of procedure. In patients with disease relapse, in a therapeutic setting, we initiated the treatment with 0.5–1 × 10^6^ CD3 positive cells/kg. The second dose contained 5 × 10^6^ CD3 positive cells/kg and the third dose was 1 × 10^7^ CD3 positive cells/kg. One patient was treated in a prophylactic setting, and his treatment was initiated with 0,5 × 10^6^ CD3 positive cells/kg. EBV was measured at every follow‐up visit, once every 7–14 days, and had been detected at an average viral load of 6.3 × 10^3^ copies/ml in the peripheral blood in five cases, as well as in the gastrointestinal biopsy or bronchoalveolar material in two cases and one case respectively. The interval from DLI application to EBV reactivation was 52 days (range: 20–83) from the first, and 30 (range: 7–65) days after the last DLI, suggesting a faster EBV reactivation associated with higher doses of DLIs. EBV reactivation regressed in all patients, spontaneously or after B‐cell depletion with the anti‐CD20 monoclonal antibody Rituximab, in four patients each. We have observed no case of PTLD post‐DLI. The occurrence of EBV reactivations associated with any of the examined patient or transplantation characteristics was not statistically significant (Table 1).

**TABLE 1:** Patient characteristics

	EBV reactivation		No EBV reactivation		
Characteristic	*N*	%	*N*	%	*P*
**Sex**					
Female	3	37.5	55	38.46	
Male	5	62.5	88	61.54	0.957
**Age at HSCT (years)**	53.11		53.18		0.987
**Diagnosis**					
Myeloid origin	3	37.5	77	53.85	
Lymphoid origin	5	62.5	66	46.15	0.476
**Conditioning**					
Myeloablative	2	25	37	25.87	
Nonmyeloablative	6	75	106	74.13	0.956
**Use of ATG**					
Yes	8	100	120	83.92	
No	0	0	23	16.08	0.608
**Donor origin**					
Related	3	37.5	47	32.86	
Unrelated	5	62.5	96	67.14	0.717
**Donor–recipient match**					
Matched	6	75	125	87.42	
Mismatched	2	25	18	12.58	0.289
**Stem cell source**					
Bone marrow	0	0	7	4.89	
Mobilized blood	8	100	136	95.11	1.000
**Prior Rituximab**					
Yes	2	25	33	23.08	
No	6	75	110	76.92	1.000
**Indication for DLI**					
Prophylaxis	1	12.5	19	13.28	
Treatment	7	87.5	124	86.72	0.949
**Prior high‐grade GVHD**					
Yes	1	12.5	16	11.19	
No	7	87.5	127	88.81	1.000
**Time from HSCT to DLI**					
>1 year	3	37.5	75	52.45	
≤1 year	5	62.5	68	47.55	1.000
**Number of DLIs**					
1 or 2	4	50	53	37.06	
3 or more	4	50	80	62.94	0.484
**CD3+ cell dose (total)**					
<50x10^6^	6	75	98	68.53	
≥50x10^6^	2	25	45	31.47	1.000

EBV via transfusion of blood products has been described, especially in immunocompromised patients [2, 3, 4, 5]. Of note, the number of B lymphocytes in a single DLI infusion is more than 1000 times higher compared with a single red blood cell concentrate, increasing the risk of both virus transmission and the establishment of a B‐cell reservoir for EBV maintenance in the immunocompromised patient.

Importantly, beyond their well‐established anti‐tumor effects [1], DLIs have been successfully used for the treatment of both EBV reactivation and post‐transplant lymphoproliferative disorder [6, 7, 8, 9]. O'Reilly et al. reported that the adoptive transfer of peripheral blood‐derived or in vitro expanded EBV‐specific T cells induced durable regressions in advanced EBV lymphomas in 16 of 18 patients [7]. The efficacy of DLIs and EBV‐specific cytotoxic T lymphocytes (CTLs) in the treatment of both previously untreated and Rituximab‐resistant EBV‐associated PTLD was confirmed by Doubrovina et al [8]. In this study, 73% of the patients treated with DLIs (*n* = 30) and 68% of those treated with EBV‐CTLs (*n* = 19) achieved sustained complete response. Similarly, in a further case series, complete responses were achieved in two patients with Rituximab‐refractory PTLD who were treated with unselected donor‐derived DLIs [9]. We speculate that in our series the DLI may have transferred EBV through B‐cell expansion in the immunocompromised environment of the recipient. At the same time, the DLI will also have transferred EBV‐specific donor T cells, allowing the self‐limitation of the viral reactivation in four of eight cases, as well as Rituximab‐supported control in the remaining four cases. Importantly, all of our patients have received ATG as part of a conditioning regiment which may have increased the risk of reactivation.

In summary, EBV reactivation after a DLI is a rare event, mostly self‐limited or feasibly controlled by an anti‐CD20 antibody treatment.

## CONFLICT OF INTEREST STATEMENT

The authors declare no conflict of interest.

## FUNDING INFORMATION

This research received no specific grant from any funding agency in the public, commercial, or not‐for‐profit sectors.

## ETHICS STATEMENT

This analysis was approved by the University Medicine Goettingen (UMG) ethics committee (Approval Number: 29/9/22)

## PATIENT CONSENT STATEMENT

Informed consent was obtained from all participants in the study.

## Data Availability

The datasets generated during and/or analyzed during the current study are available from the corresponding author upon reasonable request. The data are not publicly available due to privacy or ethical restrictions.
